# Socio-economic position and cardiovascular risk in rural indian adolescents: evidence from the Andhra Pradesh children and parents study (APCAPS)

**DOI:** 10.1016/j.puhe.2014.06.005

**Published:** 2014-09

**Authors:** S. Kinra, M. Johnson, B. Kulkarni, K.V. Rameshwar Sarma, Y. Ben-Shlomo, G.D. Smith

**Affiliations:** a Department of Epidemiology and Population Health, London School of Hygiene & Tropical Medicine, London, United Kingdom; b Cegedim Strategic Data Medical Research Ltd, London, United Kingdom; c Clinical Division, National Institute of Nutrition, Hyderabad, India; d Education and Training Division, National Institute of Nutrition, Hyderabad, India; e School of Social and Community Medicine, University of Bristol, Bristol, United Kingdom; f MRC Centre for Causal Analyses in Translational Epidemiology, School of Social and Community Medicine, University of Bristol, Bristol, United Kingdom

**Keywords:** Socio-economic position, Cardiovascular risk, Adolescents, Indian, Fat mass index, Cohort studies, BMI, body mass index, SLI, Standard of Living Index, Aix, augmentation index, ICC, intra-class correlation coefficient, HDL, high density lipoprotein, LDL, low density lipoprotein

## Abstract

**Objectives:**

This study examined association between socio-economic position and cardiovascular risk factors in adolescents to investigate whether childhood socio-economic position is a risk factor for future cardiovascular disease, independently of adult behaviours.

**Study design and methods:**

Participants (*n* = 1128, 46% girls, aged 13–18 years) were members of a birth cohort (Andhra Pradesh Children and Parents Study or APCAPS) established to investigate long-term effects of a pregnancy and childhood nutritional supplementation trial conducted in 29 villages near Hyderabad in South India. Cross-sectional associations between socio-economic position and cardiovascular risk factors were examined using linear regression models.

**Results:**

The mean BMI was 16.7 kg/m^2^ for boys and 17.8 kg/m^2^ for girls. Socio-economic position was positively associated with fat mass index (0.15 kg/m^2^; 95% CI: 0.05–0.25) and inversely associated with central-peripheral skinfold ratio (−0.04; 95% CI: −0.06 to −0.01) and, in boys, fasting triglycerides (−0.05; 95% CI: −0.09 to −0.01). Association of socio-economic position with other risk factors (blood pressure, arterial stiffness, fasting glucose, insulin and cholesterol) was weak and inconsistent, and did not persist after adjustment for potential confounders, including age, sex, pubertal stage, height, adiposity and nutrition supplementation.

**Conclusions:**

The study thus showed that lower socio-economic position may be associated with greater central adiposity and higher triglyceride levels in these settings. Socio-economic gradient in cardiovascular risk may strengthen in future with later economic and lifestyle changes. Cardiovascular disease prevention strategies should therefore focus on the youth from the low income group.

## Introduction

Based on the ecologic studies carried out in 1970s in Norway, Forsdahl hypothesised that low socio-economic position in childhood may be an independent risk factor for arteriosclerotic heart disease in later life.^1^ Age adjusted mortality rates from heart disease in adults were higher in the Norwegian counties that had experienced high infant mortality in the past, and this relationship could not be explained by the differences in living standards in later life. Since then, several studies have suggested that low socio-economic position during childhood is associated with an increased risk of mortality and morbidity related to cardiovascular disease in later life.^2^, ^3^, ^4^ The long term effect of under-nutrition in early life is one of the proposed mechanisms for this association.^5^, ^6^, ^7^

Children from the low socio-economic group tend to remain in the low socio-economic group into adulthood.^8^ Observational studies have shown that the low socio-economic position in adults is associated with behaviours such as smoking and high fat diets that may increase the risk of cardiovascular disease.^9^, ^10^ Studies investigating the relationship of socio-economic position in early life with the cardiovascular risk in adulthood have not been able to address the problem of confounding or mediation due to adult behaviour. One of the ways to disentangle the independent effect of childhood socio-economic position on later cardiovascular risk is to study these relationships pre-adulthood i.e. during childhood and adolescence.

A few studies from high-income settings have investigated the association between socio-economic position and cardiovascular risk in children and adolescents, and found an inverse association between socio-economic position and adiposity, but no clear association was observed with other cardiovascular risk factors such as blood pressure and serum cholesterol.^11^ Still fewer studies have been carried out in populations in which under-nutrition is prevalent and results from these have also been inconsistent. A study from the Democratic Republic of Congo indicated that children from the low socio-economic group had higher blood pressure than those from high socio-economic group.^12^ In some studies, under-nutrition associated with low socio-economic position has been associated with adverse cardiovascular risk.^13^ On the other hand, no independent association was observed between socio-economic position and blood pressure in Jamaican children.^14^

Assessment of the association between socio-economic position and cardiovascular risk would help focus the interventions to a vulnerable group and could inform the policy for the mitigation of cardiovascular risk. The authors therefore examined the association between socio-economic position and cardiovascular risk in a cohort of rural adolescents from South India.

## Methods

The association between socio-economic position and cardiovascular risk was examined in a rural birth cohort, Andhra Pradesh Children and Parents Study or APCAPS, established to assess the long-term impact of a nutrition supplementation trial. Cohort profile and details of the initial trial have been reported earlier.^15^, ^16^ In brief, the initial trial was conducted in 29 villages near Hyderabad, India (1987–90), using an opportunity afforded by stepwise expansion of a nutritional supplementation program (Integrated Child Development Services (ICDS) scheme). In intervention villages (*n* = 15) only, a nutritional supplement (a freshly cooked preparation made of corn–soya blend and soybean oil) was available daily to all pregnant and lactating women and children < 6 years, providing on average 2.09 MJ and 20–25 g protein to women and 1.25 MJ and 8–10 g protein to children. All births taking place in 29 villages (15 intervention, 14 control) over this time period were eligible for inclusion in the cohort. Children born in this cohort were traced in 2003–05 and invited to undergo clinical examination. This report is based on cross-sectional analyses of data collected during this follow-up.

### Measurements

An interviewer-administered questionnaire was used to collect sociodemographic, health and lifestyle information. Socio-economic position was assessed by the Standard of Living Index (SLI), which is a household-level, asset-based scale devised for use in India.^17^ The SLI has 29-items (e.g. quality of housing, toilet facilities, source of lighting and drinking water, land and animal ownership, and possession of material goods such as radio, television, bicycle, car, tractor, refrigerator, etc); a higher SLI indicates greater material affluence. The SLI is particularly suitable for use in rural India, where the joint family structure and subsistence economy renders an individual's own income a problematic measure.^18^ SLI has been widely used in epidemiological studies from India^19^ and was found to correlate highly with income data.^20^ Data on village population from the village heads, as an index of village urbanization has been collected additionally.^21^

Height was measured with a portable stadiometer (Leicester height measure; Chasmors Ltd, London, UK). The participant stood erect with the head in the Frankfort plane, and a gentle upward pressure applied under the mastoid. Weight was measured with a digital weighing machine (HD 305; Tanita, Tokyo, Japan). Skinfold thickness was measured in triplicate at four sites (biceps, triceps, subscapular and suprailiac) with a Holtain caliper (supplied by Chasmors Ltd, London, UK). Sexual maturation was classified into four stages (corresponding to Tanner's early, middle, late and postpuberty) on the basis of time since the onset of menstruation (girls) and testicular volume (boys).^22^ Testicular volume was self-assessed by children in private, using Prader's orchidometer (chain of 12 wooden testes, volumes ranging from 1 to 25 ml).

Blood pressure (BP) was measured with a validated oscillometric device (HEM 705; Omron, Matsusaka Co., Japan) in the supine position using appropriate cuff sizes. Two measurements were taken and averaged for analyses. Ambient room temperature was measured by a digital thermometer. Augmentation index (AIx), a measure of global arterial stiffness, was assessed by applanation tonometry technique using the Sphygmocor apparatus (Vx system; Atcor (PWV) Medical, Sydney, Australia). The characteristic pressure waveform produced by blood flow in the arteries changes as the arteries get stiffer with age or under conditions that lead to their premature stiffening (such as atherosclerosis).^23^ Pulse wave analysis can therefore be used to assess the functioning of the vascular tree. Pressure waveforms are obtained non-invasively by applying a pressure-sensitive probe over a peripheral artery, and transformed into the corresponding central arterial waveform by using a generalised transfer function validated against invasive pressure recordings. AIx (difference between the first and second peaks of the central arterial waveform, expressed as a percentage of pulse pressure) is negative in healthy young adults, but becomes increasingly positive as arteries stiffen.^24^ AIx was measured over the radial artery in the supine position, taking an average of two high quality recordings (quality index > 90%).

Fasting blood samples (at least 8 h) were collected in appropriate vacutainers, transferred within 1–2 h (in icebox at 4–8° Celsius) and processed within 4 h. Glucose, triglycerides, total cholesterol and high density lipoprotein (HDL) cholesterol assays were done on the same day, while insulin assays were conducted in batches within 4–6 weeks. Glucose, triglycerides, total cholesterol and HDL cholesterol were estimated with an autoanalyser (ACE Clinical System; Schiapparelli biosystems, New Jersey, US) using the recommended kits (Alfa Wasserman, New Jersey, US). Insulin was estimated by radioimmunoassay.^25^

### Quality control

Standardisation of the data collection was achieved through detailed protocols and regular training sessions, and anthropometric equipment was calibrated daily. Only one observer carried out each measurement to eliminate inter-observer bias. Internal and external quality control arrangements (with the Cardiac Biochemistry laboratory at the All India Institute of Medical Sciences, Delhi, which is part of UKNEQAS system coordinated from Newcastle, UK) were put in place for biochemical assays, and split assays were performed on a sub-sample (5%). Reproducibility of clinic measurements was assessed by repeating the measurements on a random sub-sample (5%) of participants after 1–3 weeks, and was found to be consistently high, with intra-class correlation coefficients (ICCs) of >0.98 (anthropometric assessments); >0.85 (BP and AIx); and >0.94 for biochemical assays. The average number of participants from each village was 40 (range: 2–122). ICCs for village-level clustering of outcome measures were less than 0.1, except for fasting blood glucose (ICC = 0.14). The testicular self-assessment technique was validated against a trained observer in a separate sub-study conducted in a local school, and found to be highly accurate: mean difference in model ranks (self reported minus directly observed) was 0.07 (95% CI: −0.11–0.25), with no evidence of systematic bias on Bland–Altman plot.

### Statistical analyses

The log of the sum of four skinfolds was used to calculate the percentage of body fat^26^ which was converted to fat and fat free mass using bodyweight, and expressed as relevant indices by dividing with the square of height in metres.^27^ Central adiposity was assessed by the ratio of central (subscapular + suprailiac) to peripheral (biceps + triceps) skinfolds. Low density lipoprotein (LDL) cholesterol was estimated from triglycerides and total and HDL cholesterol by using the Friedewald–Fredrickson equation.^28^ Insulin resistance was calculated by homoeostasis model assessment (HOMA), excluding those with fasting glucose ≥ 7 mmol/l.^29^ As recommended, the SLI was used to classify the participants as low (0–14), medium (15–24) and high (25–67) socio-economic position, as recommended.^17^ Urbanisation of the villages was assessed by population size (persons) classified into low (<2000 people), medium (2000–5000) and high (>5000). Suitable transformations were applied to outcome variables deviating markedly from a normal distribution.

Differences in participant characteristics in the three SLI groups were assessed with one-way analysis of variance (ANOVA) for continuous variables and Chi-squared tests for categorical variables with appropriate degrees of freedom. Weighted ANOVAs were used to account for heterogeneity of variances where appropriate. Associations between socio-economic position and cardiovascular risk factors were examined using linear regression models. Three models were fitted in each case incrementally adjusting for potential confounders or intermediaries: model 1 – age, sex and nutritional supplementation (intervention or control group); model 2 – as in model 1, plus height; and model 3 – as in model 2, plus pubertal stage, fat mass index and central-peripheral skinfold ratio. BP and AIx were additionally adjusted for ambient room temperature and heart rate, respectively, as they can artefactually affect their values.^30^, ^31^ Statistical evidence for an interaction between socio-economic position and sex was sought, and where evidence was supportive, associations were examined separately for boys and girls. Robust standard errors were used throughout to account for village-level clustering of the data. All statistical analyses were carried out using STATA version 10 (StataCorp, College Station, Texas).

Sample size calculations for the main study were carried out for the primary outcomes of cardiovascular risk. The study was adequately powered (80% power at 5% significance level and 0.01 ICC for village-level clustering) to detect mean differences of 1.8–2.9 mmHg for systolic BP, 1.5% for Aix and 1.7–3.2 mU/ml for fasting insulin.

## Results

At the follow-up assessment, 1963 (71%) families could be traced successfully to the original trial. Of the 2601 children eligible for study inclusion (i.e. born to these families during 1987–90 and still alive in 2002–05), only those with baseline information from the trial (*n* = 1492; 57%) were invited for clinical examination. A total of 1165 children participated in the clinics: 654 (82%) in the intervention and 511 (74%) in the control area, representing 45% (49% intervention, 41% control) of all births during the trial period.^15^ Children who took part in the clinics were similar to those who were eligible but did not participate. After excluding three pregnant girls, there were 1162 participants. Complete data on anthropometric measures and relevant confounders were available for 1128 (97%) adolescents. The median age of the participants at the follow-up assessment was 15.7 years (range: 13–18 years), of which 54% were boys. Overall, the participants were of relatively short stature (boys: 158.8 cm; girls: 151.5 cm) with a low body mass index (BMI) (boys: 16.7 kg/m^2^; girls: 17.8 kg/m^2^) which is consistent with a chronically undernourished population. Only two children reported tobacco use or alcohol consumption, so these variables were not considered in the analyses. Thirty five per cent of children belonged to the high SLI group, 47% belonged to the middle SLI group and 18% children belonged to the low SLI group. The distribution of SLI was consistent with the peri-urban situation of these villages.

Table 1 indicates participant characteristics in the three SLI groups of girls and boys. The proportion of individuals in the three SLI groups was different among the boys and girls with a smaller proportion (<10%) of girls belonging to the low SLI group. More than 50% of girls were postpubertal as expected due to an earlier onset of puberty in girls, whereas none of the boys were postpubertal. As expected, boys and girls from the higher SLI groups were taller than their counterparts in the lower SLI groups. In the case of boys, but not the girls, higher socio-economic position was associated with higher BMI and fat mass index. Central-peripheral skin fold ratio, however, was lower in the high SLI group, in boys and girls. In the case of boys, blood pressure and serum insulin were higher, and AIx and serum triglycerides were lower in the high SLI group, but these differences were not observed in girls.

**Table 1:** Participant characteristics by Standard of Living Index (SLI), ÁPCAPS follow-up, 2003–05.

	Boys (*N* = 607)					Girls (*N* = 521)				
	*N*	Low SLI (*N* = 164)	Middle SLI (*N* = 281)	High SLI (*N* = 162)	*P*-value*	*N*	Low SLI (*N* = 48)	Middle SLI (*N* = 249)	High SLI (*N* = 224)	*P*-value*
**Sociodemographics**										
Age (years)	607	15.9 (0.9)	15.9 (0.9)	15.9 (0.8)	0.967	521	15.7 (0.9)	15.8 (0.9)	15.8 (1.0)	0.855
Pubertal stage (*n*, %)	604				0.508	517				0.880
Early puberty	171	38 (23.5)	87 (31.0)	46 (28.6)		0	0 (0.0)	0 (0.0)	0 (0.0)	
Middle puberty	322	94 (58.0)	145 (51.6)	83 (51.6)		43	4 (8.3)	22 (8.9)	17 (7.7)	
Late puberty	111	30 (18.5)	49 (17.4)	32 (19.9)		204	17 (35.4)	94 (38.1)	93 (41.9)	
Postpuberty	0	0 (0.0)	0 (0.0)	0 (0.0)		270	27 (56.3)	131 (53.0)	112 (50.5)	
**Anthropometry**										
Height (mm)	604	1580.5 (81.5)	1579.3 (86.7)	1613.1 (82.7)	<0.001	516	1495.0 (58.8)	1516.4 (55.4)	1517.3 (58.1)	0.040
Body mass index (kg/m^2^)	604	16.5 (1.8)	16.5 (1.8)	17.2 (2.6)	<0.001**	516	17.8 (2.2)	17.7 (1.8)	18.0 (2.6)	0.254**
Fat mass index (kg/m^2^)	604	1.5 (0.5)	1.6 (0.6)	1.8 (1.0)	<0.001**	516	3.7 (1.0)	3.7 (0.9)	3.8 (1.3)	0.226**
Central-peripheral skinfold ratio	604	1.6 (0.2)	1.5 (0.2)	1.5 (0.2)	0.002	516	1.5 (0.3)	1.4 (0.2)	1.4 (0.2)	0.033
**Cardio-metabolic risk**										
Systolic blood pressure (mmHg)	603	109.7 (11.1)	110.2 (10.7)	112.2 (10.9)	0.080	515	107.5 (8.3)	107.5 (9.3)	107.2 (9.2)	0.917
Diastolic blood pressure (mmHg)	603	61.4 (6.8)	61.7 (6.5)	63.8 (6.4)	0.001	515	63.0 (5.7)	62.6 (7.0)	62.7 (6.0)	0.924
Augmentation index (%)	486	5.1 (10.4)	3.8 (9.8)	1.8 (10.9)	0.035	376	6.8 (12.3)	3.9 (11.1)	4.8 (9.9)	0.365
Total cholesterol (mmol/l)	567	3.2 (0.6)	3.3 (0.7)	3.3 (0.6)	0.769	483	3.5 (0.7)	3.6 (0.7)	3.7 (0.7)	0.040
LDL cholesterol (mmol/l)	567	1.9 (0.5)	1.9 (0.6)	2.0 (0.5)	0.577	483	2.1 (0.7)	2.2 (0.6)	2.2 (0.6)	0.247
HDL cholesterol (mmol/l)	567	1.0 (0.2)	1.0 (0.2)	1.0 (0.2)	0.146	483	0.9 (0.3)	1.0 (0.2)	1.0 (0.2)	0.093
Triglycerides^a^ (mmol/l)	567	0.8 (1.4)	0.8 (1.4)	0.7 (1.4)	0.036	483	0.9 (1.5)	0.9 (1.4)	0.9 (1.5)	0.305
Glucose (mmol/l)	543	4.7 (0.7)	4.7 (0.6)	4.7 (0.6)	0.828	465	4.7 (0.6)	4.8 (0.8)	4.6 (0.6)	0.027**
Insulin^a^ (mU/l)	543	16.4 (1.7)	15.2 (1.8)	17.9 (1.8)	0.020	465	16.6 (1.8)	16.7 (1.8)	18.1 (1.7)	0.275
HOMA score^a^	541	3.4 (1.7)	3.1 (1.8)	3.7 (1.8)	0.022	462	3.4 (1.8)	3.4 (1.9)	3.7 (1.7)	0.474

LDL: low density lipoprotein; HDL: high density lipoprotein; HOMA: homoeostasis modal assessment.

Unless stated otherwise, values are means (standard deviations).

* *P*-values are based on one-way analysis of variance (ANOVA) and Chi-squared tests with appropriate degrees of freedom.

** *P*-values are based on weighted ANOVAs to account for heterogeneity of variances.

a Geometric means and geometric standard deviations as factor on logarithmic scale.

Table 2 presents the adjusted differences in cardiovascular risk factors across the SLI groups. All models were adjusted for sex except triglycerides, for which there was statistical evidence of an interaction between socio-economic position and sex (*P*_interaction_ = 0.008); hence, the models for triglycerides are presented separately for boys and girls. Socio-economic position was positively associated with fat mass index (0.15 kg/m^2^; 95% CI: 0.05–0.25) and inversely associated with central-peripheral skinfold ratio (−0.04; 95% CI: −0.06 to −0.01) and, in boys only, fasting triglycerides (−0.05; 95% CI: −0.09 to −0.01). Associations with other risk factors (blood pressure, arterial stiffness, fasting glucose, insulin and cholesterol) were weak and inconsistent, and did not persist after adjustment for potential confounders, including age, sex, pubertal stage, height, adiposity and nutrition supplementation.

**Table 2:** Association between Standard of Living Index and cardiovascular risk among participants of the ÁPCAPS follow-up, 2003–05.

	Model 1			Model 2			Model 3		
	*β* Coeff	95% CI	*P*-value	*β* Coeff	95% CI	*P*-value	*β* Coeff	95% CI	*P*-value
Fat mass index (kg/m^2^)	0.15	0.05 to 0.25	0.01	0.13	0.04 to 0.23	0.01	0.15	0.05 to 0.25	0.01^a^
Central-peripheral skinfold ratio	−0.04	−0.06 to −0.01	0.01	−0.05	−0.07 to −0.02	<0.01	−0.05	−0.07 to −0.03	<0.01^b^
Systolic blood pressure (mmHg)	0.55	−0.23 to 1.33	0.16	0.09	−0.61 to 0.78	0.80	0.15	−0.58 to 0.87	0.68
Diastolic blood pressure (mmHg)	0.54	0.07 to 1.00	0.03	0.40	−0.07 to 0.87	0.09	0.38	−0.08 to 0.84	0.10
Augmentation index (%)	−1.21	−2.36 to −0.06	0.04	−0.72	−1.84 to 0.40	0.20	−0.63	−1.72 to 0.45	0.24
Total cholesterol (mmol/l)	0.06	−0.01 to 0.13	0.06	0.07	0.01 to 0.13	0.04	0.05	−0.01 to 0.11	0.10
LDL cholesterol (mmol/l)	0.05	−0.01 to 0.10	0.09	0.05	−0.01 to 0.11	0.08	0.03	−0.02 to 0.08	0.20
HDL cholesterol (mmol/l)	0.02	−0.01 to 0.04	0.06	0.02	0.01 to 0.04	0.04	0.02	−0.01 to 0.04	0.06
Triglycerides^c^ (mmol/l) in boys^d^	−0.05	−0.09 to −0.01	0.02	−0.04	−0.09 to −0.01	0.03	−0.04	−0.08 to −0.01	0.02
Triglycerides^c^ (mmol/l) in girls^d^	−0.04	−0.02 to 0.11	0.17	0.05	−0.02 to 0.11	0.14	−0.05	−0.02 to 0.11	0.17
Glucose (mmol/l)	−0.04	−0.12 to 0.04	0.29	−0.04	−0.12 to 0.04	0.29	−0.03	−0.10 to 0.04	0.35
Insulin^c^ (mU/l)	0.06	0.01 to 0.11	0.05	0.05	−0.01 to 0.10	0.10	0.05	−0.01 to 0.10	0.07
HOMA score^c^	0.05	−0.01 to 0.11	0.09	0.04	−0.02 to 0.10	0.16	0.05	−0.01 to 0.11	0.11

LDL: low density lipoprotein; HDL: high density lipoprotein; HOMA: homoeostasis modal assessment.

*β* coeff: *β* coefficients are the mean differences across categories of the Standard of Living Index (i.e. average difference between the middle SLI – low SLI and high SLI– middle SLI categories). Positive difference indicates a higher value in the higher Standard of Living Index category.

Model 1: adjusted for age, gender (except triglyceride models), nutritional supplementation, room temperature (blood pressure only) and heart rate (augmentation index only).

Model 2: adjusted for variables in model 1 + height.

Model 3: adjusted for variables in model 2 + pubertal stage, fat mass index and central-peripheral skinfold ratio.

Sample size: *n* = 1120 for fat mass index and central-peripheral skin fold ratio; *n* = 1118 for systolic and diastolic blood pressure; *n* = 862 for augmentation index; *n* = 1050 for total cholesterol, LDL cholesterol and HDL cholesterol; *n* = 567 for triglycerides in boys and *n* = 483 for triglycerides in girls; *n* = 1008 for glucose and insulin; and *n* = 1003 for HOMA score.

a Not adjusted for fat mass index.

b Not adjusted for central-peripheral skinfold ratio.

c Differences between means are on log scale. On original scale these equate to (for model 1): triglycerides in boys 0.95 (0.91–0.99), triglycerides in girls 1.04 (0.98–1.12), insulin 1.06 (1.00–1.12) and HOMA score 1.05 (0.99–1.12).

d There was no strong evidence of interaction between Standard of Living Index and sex for any of the outcome measures except triglycerides (*P*_interaction_ = 0.008), for which data are presented separately for boys and girls.

## Discussion

In this study in a rural adolescent cohort, lower socio-economic position was associated with central adiposity and higher serum triglyceride levels. However, no clear associations were observed between socio-economic position and other cardiovascular risk factors.

Studies in adults from high-income countries have generally found an inverse association between socio-economic position and cardiovascular risk.^32^, ^33^ On the other hand, contrary to a more consistent pattern noted in adults, studies in children and adolescents in these settings have found inconsistent associations between socio-economic position and cardiovascular risk assessed using different indicators of cardiovascular risk such as adiposity, blood pressure, lipid profile, C-reactive protein and homocysteine levels.^34^, ^35^, ^36^, ^37^ Studies from low-income countries such as India have also shown inconsistent results in the case of adults as well as adolescents, finding both direct and inverse associations between measures of socio-economic position and cardiovascular risk.^38^, ^39^ Findings from this study corroborate the inverse association between socio-economic position and cardiovascular risk as central adiposity and higher triglyceride levels could be considered as early indicators of enhanced cardiovascular risk in this population.

A number of prospective studies have shown that adiposity, especially central adiposity in childhood and adolescence is linked to adverse cardio-metabolic risk profile in later life.^40^, ^41^ Data from the Avon Longitudinal Study of Parents and Children (ALSPAC) indicated that adiposity in childhood was associated with increased cardiovascular risk in later life.^42^ A study from Denmark also showed that high plasma triglyceride and high BMI in childhood were associated with low insulin-sensitivity index values in young adulthood.^43^ It may be speculated that the inverse relationship between socio-economic status and cardiovascular risk factors may become more pronounced at a later stage in the participants of the present study.

Low socio-economic position increases cardiovascular disease risk through a number of unfavourable environmental exposures including early under nutrition leading to developmental programming of adult health. In addition, chronic inflammation associated with higher rates of infections in low income group populations as well as psychological stress have been implicated as reasons for the inverse association of cardiovascular risk with socio-economic position. Cereal based high carbohydrate diets commonly consumed by low income group populations in India are also known to be associated with higher triglyceride levels.^44^ Apart from these environmental influences, low socio-economic status could also enhance the disease risk through behavioural risk factors as adolescents from the low-income group are more likely to engage in risk behaviours such as smoking and physical inactivity.^34^ The inverse association of socio-economic position with cardiovascular disease risk in the present study is, however, unlikely to be influenced by socio-economic differences in behavioural risk factors. The participants were lean irrespective of their socio-economic position indicating an absence of urbanisation and related lifestyle changes at this stage. Behavioural risk factors such as smoking were almost absent in this study's participants as young adults in India start smoking at a relatively later age. With lifestyle changes consequent to future urbanization of these settings, association between socio-economic position and cardiovascular risk may strengthen at a later stage.

### Strengths and limitations

To the knowledge of authors, this is one of the few studies from a developing country setting that has assessed socio-economic gradient in relation to the cardiovascular risk factors in a young cohort. In addition, the study assessed a wide range of cardiovascular risk factors and therefore a comprehensive assessment of the relationship between the socio-economic position and cardiovascular risk was possible. An important limitation of this study was the narrow range of socio-economic differences in this rural cohort which may have contributed to the inability to detect differences in cardiovascular risk factors due to reduced study power. The observation that more boys came from poorer households suggests some sort of selection bias may have operated, as parents in low castes may be less likely to let their girls attend, even though the overall response rate amongst those who were invited is relatively high. Alternatively, since girls in the lower SLI groups in India get married at a younger age, some may have moved out of the study area. Another limitation was the loss to follow-up in the cohort which could potentially bias the findings if associated with the socio-economic position of the participants. However, the baseline characteristics of the participants and non-participants were not markedly different.^15^, ^16^

### Conclusions

The present study suggests an inverse relationship between socio-economic position and cardiovascular disease risk in this cohort of rural Indian adolescents. Cardiovascular risk mitigation strategy should therefore focus on the youth belonging to the low socio-economic stratum.

## Author statements

### Ethics approval

Ethical approval for the study was obtained from the ethics committee of the National Institute of Nutrition, Hyderabad and the study was performed in accordance with the ethical standards laid down in the Declaration of Helsinki. Approval was also sought from the village heads and their committees in each of the 29 villages. Written informed consent (witnessed thumbprint if illiterate) was obtained from the participants and their parents (or guardians) prior to their inclusion in the study. The study sponsor played no part in the design, collection or analysis of data, interpretation of findings, writing of the manuscript or the decision to submit it for publication.

### Funding

The initial trial was funded jointly by the Indian Council of Medical Research and the United States Assistance for International Development. The follow-up study was funded by a personal fellowship to SK (Eden Fellowship in Paediatrics, granted by the Royal College of Physicians, UK).

### Conflict of interest

All authors declare that they had no financial or personal relationships with study sponsors or other people or organisations that could inappropriately influence (bias) their work.
